# Expression of Claudin-18.2 in pancreatic ductal adenocarcinoma: a systematic review and meta-analysis

**DOI:** 10.3389/fmed.2026.1809374

**Published:** 2026-04-24

**Authors:** Mohammed Al Subhi, Deema Al-Nabhani, Maryam Al Sarmi, Ahmed Abd Elrahman, Yaqoob AlSawafi, Srinivasa Rao Sirasanagandla, Younis Al-Mufargi

**Affiliations:** 1 School of Medicine, Medical Sciences and Nutrition, University of Aberdeen, Foresterhill, Aberdeen, Scotland, United Kingdom; 2 School of Medicine, Dentistry and Nursing, College of Medical, Veterinary and Life Sciences, University of Glasgow, Glasgow, United Kingdom; 3 Royal College of Surgeons in Ireland (RCSI), University of Medicine and Health Sciences, Dublin, Ireland; 4 Department of General Surgery, Medical City for Military and Security Services, Muscat, Oman; 5 Department of Human and Clinical Anatomy, College of Medicine and Health Sciences, Sultan Qaboos University, Muscat, Oman

**Keywords:** biomarker, Claudin-18.2, CLDN18.2, immunohistochemistry, meta-analysis, pancreatic ductal adenocarcinoma, zolbetuximab

## Abstract

**Background:**

Claudin-18.2 (CLDN18.2), a tight junction protein, has emerged as a promising molecular target in gastrointestinal cancers. However, its expression pattern and clinicopathological relevance in pancreatic ductal adenocarcinoma (PDAC) remain unclear.

**Objective:**

To assess the prevalence, tumor-specific, and clinicopathological associations of CLDN18.2 expression in PDAC, and to identify sources of heterogeneity to clarify its value as a therapeutic target.

**Methods:**

A systematic search across six major databases was conducted from inception to August 2025 in accordance with PRISMA guidelines. Included studies evaluated CLDN18.2 expression in adult PDAC using defined immunohistochemistry protocols. Random-effects models estimated pooled prevalence and odds ratios (ORs), with subgroup analyses exploring heterogeneity.

**Results:**

Sixteen studies including 2,025 patients with PDAC were included. The pooled prevalence of CLDN18.2 expression was 51.60% (95% CI: 40.93–62.19), with substantial heterogeneity (I^2^ = 95.4%). Expression varied significantly by antibody clone (*p* = 0.0006), but not by geographic regions. CLDN18.2 expression was highly specific to neoplastic tissue (OR = 102.40; 95% CI: 35.50–295.38). No significant associations were identified with sex, tumor location, T-stage, N-stage, or metastatic status. However, expression was significantly lower in poorly differentiated tumors (G3 vs. G1/G2: OR = 0.37; 95% CI: 0.20–0.70).

**Conclusion:**

CLDN18.2 is frequently expressed in PDAC and shows high tumor specificity, supporting its relevance as a therapeutic biomarker. Despite assay variability, even under stringent clinical trial criteria, a meaningful subset of patients may qualify for CLDN18.2-targeted therapy. These findings highlight the need for standardized testing and further clinical evaluation of CLDN18.2-directed strategies in PDAC.

## Highlights

- CLDN18.2 is a tumor-specific biomarker in pancreatic ductal adenocarcinoma, expressed in approximately 51.6% of cases.
- Using stringent, trial-aligned immunohistochemical criteria, about 23% of PDAC cases may be eligible for CLDN18.2-targeted therapy.
- Reported CLDN18.2 expression varies widely, likely due to differences in immunohistochemical methods, underscoring the need for standardized testing.

## 1 Introduction

Pancreatic ductal adenocarcinoma (PDAC), the most common form of pancreatic cancer, remains one of the deadliest malignancies worldwide, with over 511,000 new cases and a 5-year survival rate of just 13% (1–3). It has recently become the third leading cause of cancer-related death in the United States (1). These poor outcomes are largely due to late diagnosis of the disease (45.9% are diagnosed at stage IV) (4).

The Claudin family consists of 27 transmembrane tight junction proteins. In biological systems, Claudins have been shown to function as paracellular barriers, paracellular channels, and signaling hubs for cellular tumorigenesis (5). Altered expression of Claudins has been associated with many cancers (6), including Claudin-18 in PDAC (7). Claudin-18 has two isoforms (Claudin-18.1 and Claudin-18.2), which are normally expressed in pulmonary and gastric tissue, respectively (8). CLDN18.2 has been implicated in gastric, pancreatic, esophageal, ovarian, and lung tumors (9).

In 2024, the FDA approved zolbetuximab as the first CLDN18.2-targeted treatment for gastric and gastro-esophageal adenocarcinoma (10). Zolbetuximab is a chimeric immunoglobulin G1 monoclonal antibody that binds to CLDN18.2, leading to cellular apoptosis and inhibition of proliferation (11). It has been shown to prolong progression-free survival and overall survival in both the SPOTLIGHT and GLOW trials (10, 12). Currently, there are two ongoing studies assessing the use of zolbetuximab in metastatic pancreatic cancer (NCT06396091; and NCT03816163). Moreover, five additional therapies targeting CLDN18.2 are currently under clinical investigation (NCT07079228; NCT07025889; NCT06219941; NCT05862324; and NCT05482893).

Given the involvement of CLDN18.2 in PDAC and the ongoing investigations into therapies targeting this protein, our aim to systematically synthesize the evidence on CLDN18.2 expression in PDAC through a systematic review and meta-analysis, evaluating its association with clinicopathological features and its potential as a therapeutic target in future clinical applications.

## 2 Materials and methods

### 2.1 Study design

This study was conducted as a systematic review and meta-analysis of observational studies, following the Preferred Reporting Items for Systematic Reviews and Meta-Analyses (PRISMA) guidelines. The review aimed to synthesize available evidence on CLDN18.2 expression in PDAC, its tumor specificity, and its association with clinicopathological characteristics.

### 2.2 Search strategy

We conducted a comprehensive and a systematic literature review in EBM Reviews, MEDLINE, and Embase (via Ovid), as well as in Web of Science, Scopus, and PubMed (inception to 08 of August 2025). Search terms included Claudin-18.2 and its variants (“Claudin 18,” “Claudin-18,” CLDN18.2, “Claudin 18 isoform 2,” “Claudin 18 variant 2”) and pancreatic cancer terms (“Pancreatic Ductal Adenocarcinoma,” PDAC, “Pancreatic cancer,” “Pancreatic carcinoma,” “Pancreatic neoplasm*,” “Pancreatobiliary adenocarcinoma”). Two reviewers independently screened titles, abstracts, and full texts based on predefined criteria. Discrepancies were resolved by discussion or a third reviewer. Reference lists of the included studies were reviewed manually to include relevant studies to the topic.

### 2.3 Eligibility criteria

We included studies that evaluated the expression of CLDN18.2 in adult patients with PDAC using immunohistochemistry (IHC). Eligible studies were required to report the number of CLDN18.2-positive cases using well defined IHC protocols. Studies were excluded if they involved non-PDAC pancreatic tumors, were inaccessible due to paywalls, or were case reports, abstracts, reviews, or non-original research. Additionally, studies not published in English were excluded.

### 2.4 Data extraction

Data were independently extracted by two reviewers using a standardized data extraction form. The extracted information included study characteristics (author, year, and country), sample size, number and percentage of CLDN18.2-positive cases, antibody clone used, staining protocols, positivity thresholds, and reported associations with clinicopathological features. Any discrepancies between reviewers were resolved through discussion or consultation with a third reviewer to ensure accuracy and consistency.

### 2.5 Interpretation of CLDN18.2 positivity

To harmonize definitions across studies, CLDN18.2 positivity was interpreted according to each study’s reported criteria. When quantitative thresholds (e.g., % positive cells, intensity score, or H-score) were provided, these were applied directly. In studies lacking explicit cut-offs, any membranous staining of tumor cells (≥1+) was considered positive, consistent with author reporting.

### 2.6 Quality assessment

The methodological quality of the included studies was assessed using the Joanna Briggs Institute (JBI) Critical Appraisal Checklist for Studies Reporting Prevalence Data. The checklist includes eight domains: sample frame appropriateness, sampling method, sample size adequacy, description of participants and setting, data analysis coverage, validity of condition identification, standardization of measurement, and appropriateness of statistical analysis. Each domain was rated as “Yes,” “No,” or “Unclear.” Response rate was deemed not applicable in these retrospective tissue-based studies. Studies meeting ≥6 of 8 applicable criteria, were classified as high quality, 4–5 as moderate, and ≤3 as low quality. Two independent reviewers performed the assessment, and discrepancies were resolved through discussion.

### 2.7 Statistical analysis

Meta-analyses were performed using a random-effects model (DerSimonian and Laird method) to account for expected heterogeneity across studies due to differences in patient populations, antibody clones, staining protocols, and positivity thresholds. For the primary outcome, we calculated the pooled prevalence of CLDN18.2 expression in PDAC, expressed as a percentage with corresponding 95% confidence intervals (CIs). For all dichotomous outcomes, including comparisons of expression by sex, tumor location, tumor stage (T), nodal status (N), metastatic status (M), and tumor grade (differentiation), we computed odds ratios (ORs) with 95% CIs. Where applicable, subgroup meta-analyses were conducted based on geographic region (Asia, Europe, North America) and antibody clone used in IHC (e.g., 43–14A, EPR19202, ZMD395, HPA-018446). Statistical differences between subgroups were assessed using Q-statistics for subgroup difference. Heterogeneity across studies was quantified using the I^2^ statistic, with values of 25, 50, and 75% representing low, moderate, and high heterogeneity, respectively. A *p*-value of less than 0.10 for the Cochran Q test was considered indicative of significant heterogeneity. Forest plots were generated for all pooled analyses to visually represent the effect sizes and confidence intervals. To assess publication bias, we visually examined funnel plots of the primary outcome (CLDN18.2 prevalence in PDAC). All statistical analyses and data visualizations were performed using RStudio (Version 2023.06.1 Build 524) with R (Version 4.3.1). A *p*-value < 0.05 was considered a statistically significant.

## 3 Results

**Figure 1:**
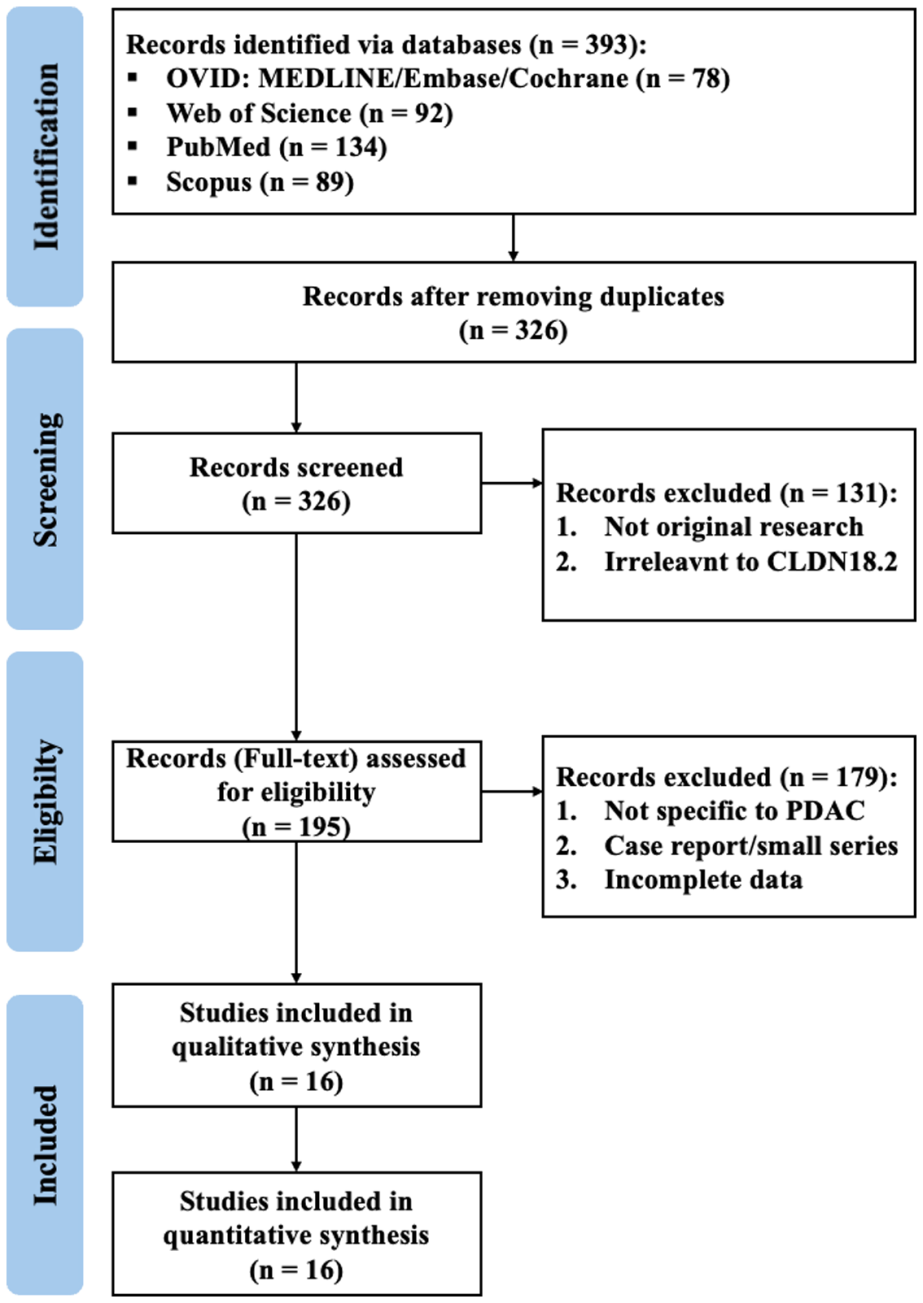
PRISMA flow diagram of study selection showing the identification, screening, eligibility assessment, and inclusion of studies. Of 393 records identified across four databases, 326 remained after duplicate removal. Following title and abstract screening and full-text review, 16 studies met the eligibility criteria for qualitative and quantitative synthesis.

Following PRISMA guidelines, 393 records were identified. After duplicate removal, 326 were screened, with 131 excluded. Of 195 full-text articles assessed, 16 studies involving 2,025 PDAC patients were included in the final analysis (Figure 1). The key characteristics and findings of the included observational studies are summarized in Table 1.

**Table 1:** Overview and characteristics of included observational studies on CLDN18.2 expression in pancreatic ductal adenocarcinoma.

Study (author, year)	Country	Patients with PDAC (n, % positive)	CLDN18.2 in neoplastic vs. non-neoplastic tissue	Positivity definition	Antibody clone; dilution; manufacturer	Key findings
Valentini et al., 2025 (22)	Italy	70 (35, 50%)	75 (35) vs. 28 (0)	H-score ≥ 5	EPR19202; 1:200; Abcam	Only neoplastic cells expressed CLDN18.2. Associated with well/moderately differentiated tumors and N0 status.
Kayikcioglu et al., 2023 (23)	Turkey	68 (37, 54.4%)	NR	Any membranous staining ≥1	Unspecified	Associated with better overall survival.
Wöll et al., 2014 (24)	Germany	174 (103, 59.2%)	202 (109) vs. 24 (0)	≥1% tumor cells stained	aGC182; NA; in-house	Correlated with lymph node metastasis.
Arseneau et al., 2025 (25)	Canada	120 (39, 32.5%)	NR	≥75% tumor cells, intensity ≥2+	43–14A; pre-diluted; Roche	Associated with well-differentiated tumors and better survival.
Park et al., 2023 (13)	South Korea	130 (41, 31.5%)	NR	≥80% tumor cells, intensity ≥2+	34H14L15; 1:100; Invitrogen	Associated with well-differentiated tumors and N0 status.
Wang et al., 2022 (15)	China	93 (46, 49.5%)	93 (35) vs. 13 (0)	H-score >150	EPR19202; 1:500; Abcam	Higher expression in PDAC than normal tissue. Linked with metastasis, stage, nerve invasion, poor survival in stage III/IV.
Lyu et al., 2024 (18)	Germany	309 (94, 30.4%)	NR	≥75% tumor cells, intensity ≥2+	LS-B16145; 1:200; LSBio & 43–14A; Roche	Associated with better differentiation and survival. Comparable clone performance.
Zhang et al., 2022 (26)	China	302 (171, 56.6%)	302 (171) vs. 10 (0)	≥1% tumor cells stained	Catalog CLDN18.2	Associated with better differentiation, female sex, non-smokers, less bile duct invasion/metastasis.
Sanada et al., 2010 (27)	Japan	15 (6, 40%)	NR	≥10% tumor cells stained	Claudin 18; 1:200; Zymed	NA
Karanjawala et al., 2008 (7)	USA	166 (83, 50%)	166 (159) vs. 105 (4)	>80% tumor cells, intensity ≥2+	ZMD395; 1:400; Invitrogen	Overexpressed in neoplastic tissue. Associated with well-differentiated tumors.
Soini et al., 2012 (28)	Japan and Finland	111 (78, 70.3%)	18 (10) vs. 26 (0)	>25% tumor cells stained	No. 38–8,000; 1:100; Invitrogen	Associated with well-differentiated tumors.
Isidro et al., 2022 (29)	USA	56 (37, 66.1%)	NR	>5% tumor cells, intensity ≥1+	HPA-018446; 1:500; Sigma-Aldrich	NA
Yang et al., 2022 (30)	Taiwan	10 (10, 100%)	All effusion samples	Staining intensity ≥1%	HPA-018446; NA; Sigma-Aldrich	NA
Tanaka et al., 2011 (31)	Japan	156 (109, 69.9%)	NR	≥1% tumor cells stained	ZMD395; 1:1000; Zymed	High expression in cancer tissue. Associated with well/moderately differentiated tumors.
Kyuno et al., 2025 (32)	Japan	201 (20, 10%)	211 (152) vs. 153 (3)	≥75% tumor cells, intensity ≥2+	43–14A; 1:5000; Abcam	High expression in neoplastic tissue. Biopsy detected 54.6% of CLDN18.2 + tumors.
Li et al., 2020 (33)	Taiwan	44 (30, 68.2%)	NR	>1 + (weak, <10% of cells)	HPA-018446; 1:250; Sigma-Aldrich	CLDN18.2 has high sensitivity as a diagnostic tool for gastric and pancreatobiliary tumors.

PDAC, Pancreatic ductal adenocarcinoma; NR, Not reported; NA, Not available; N0, No regional lymph node metastasis. Percent positivity reflects the proportion of PDAC cases expressing CLDN18.2 per study-specific criteria. Antibody information includes clone name, dilution, and manufacturer, where available. Staining thresholds vary across studies, contributing to inter-study heterogeneity.

The methodological quality of the 16 included studies was evaluated using the Joanna Briggs Institute (JBI) Critical Appraisal Checklist for Prevalence Studies. Overall, 7 studies (43.8%) were rated as high quality, and 9 studies (56.2%) as moderate quality. None were rated as low quality. The most common limitation was the lack of sample size justification and unclear or non-random sampling methods. However, all studies used valid and standardized IHC methods to assess CLDN18.2 expression. Details of the quality assessment are presented in Table 2.

**Table 2:** Quality assessment of included studies based on the JBI checklist for prevalence studies.

Study (author, year)	Q1: sampling frame	Q2: sampling method	Q3: sample size	Q4: subjects and setting described	Q5: valid method for condition	Q6: standardized measurement	Q7: appropriate analysis	Q8: adequate response rate	“Yes” total	Overall quality
Valentini et al., 2025 (22)	Yes	Yes	No	Yes	Yes	Yes	Yes	Yes	7	High
Kayikcioglu et al., 2023 (23)	Yes	Yes	No	Yes	Yes	Yes	Yes	Yes	7	High
Wöll et al., 2014 (24)	Unclear	Unclear	No	Yes	Yes	Yes	Yes	Yes	5	Moderate
Arseneau et al., 2025 (25)	Yes	Yes	No	Yes	Yes	Yes	Yes	Yes	7	High
Park et al., 2023 (13)	Yes	Unclear	No	Yes	Yes	Yes	Yes	Yes	6	High
Wang et al., 2022 (15)	Unclear	Unclear	No	Yes	Yes	Yes	Yes	Yes	5	Moderate
Lyu et al., 2024 (18)	Yes	Yes	No	Yes	Yes	Yes	Yes	Yes	7	High
Zhang et al., 2022 (26)	Yes	Yes	No	Yes	Yes	Yes	Yes	Yes	7	High
Sanada et al., 2010 (27)	Unclear	Unclear	No	Yes	Yes	Yes	Yes	Unclear	5	Moderate
Karanjawala et al., 2008 (7)	Unclear	Unclear	No	Yes	Yes	Yes	Yes	Yes	5	Moderate
Soini et al., 2012 (28)	Unclear	Unclear	No	Yes	Yes	Yes	Yes	Yes	5	Moderate
Isidro et al., 2022 (29)	Unclear	Unclear	No	Yes	Yes	Yes	Yes	Yes	5	Moderate
Yang et al., 2022 (30)	Unclear	No	No	Yes	Yes	Yes	Yes	Yes	5	Moderate
Tanaka et al., 2011 (31)	Unclear	Unclear	No	Yes	Yes	Yes	Yes	Yes	5	Moderate
Kyuno et al., 2025 (32)	Yes	Yes	No	Yes	Yes	Yes	Yes	Yes	7	High
Li et al., 2020 (33)	Unclear	Unclear	No	Yes	Yes	Yes	Yes	Yes	5	Moderate

This table summarizes the quality assessment of included studies using the Joanna Briggs Institute (JBI) Checklist for Prevalence Studies, which evaluates eight domains (Q1–Q8) related to sampling, measurement, and analysis. Each item was rated as “Yes,” “No,” or “Unclear.” Studies were categorized as high quality (6–8 “Yes” responses), moderate quality (4–5 “Yes”), or low quality (≤3 “Yes”).

**Figure 2:**
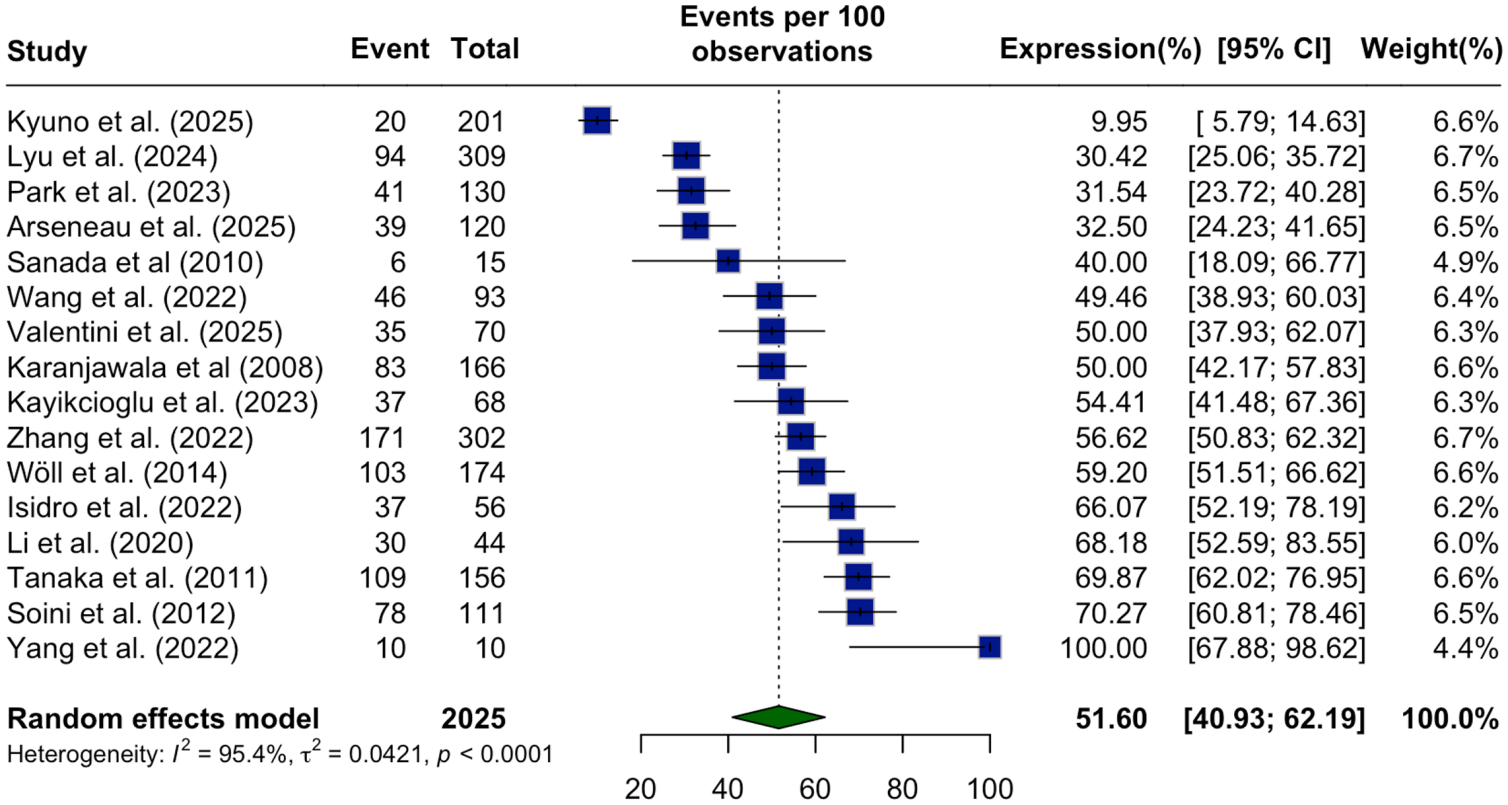
Pooled prevalence of CLDN18.2 expression in pancreatic ductal adenocarcinoma (PDAC) shown as a forest plot summarizing 16 studies comprising 2025 patients. The overall pooled prevalence was 51.60% (95% CI, 40.93–62.19) with substantial heterogeneity (*I*^2^ = 95.4%, *p* < 0.0001).

**Figure 3:**
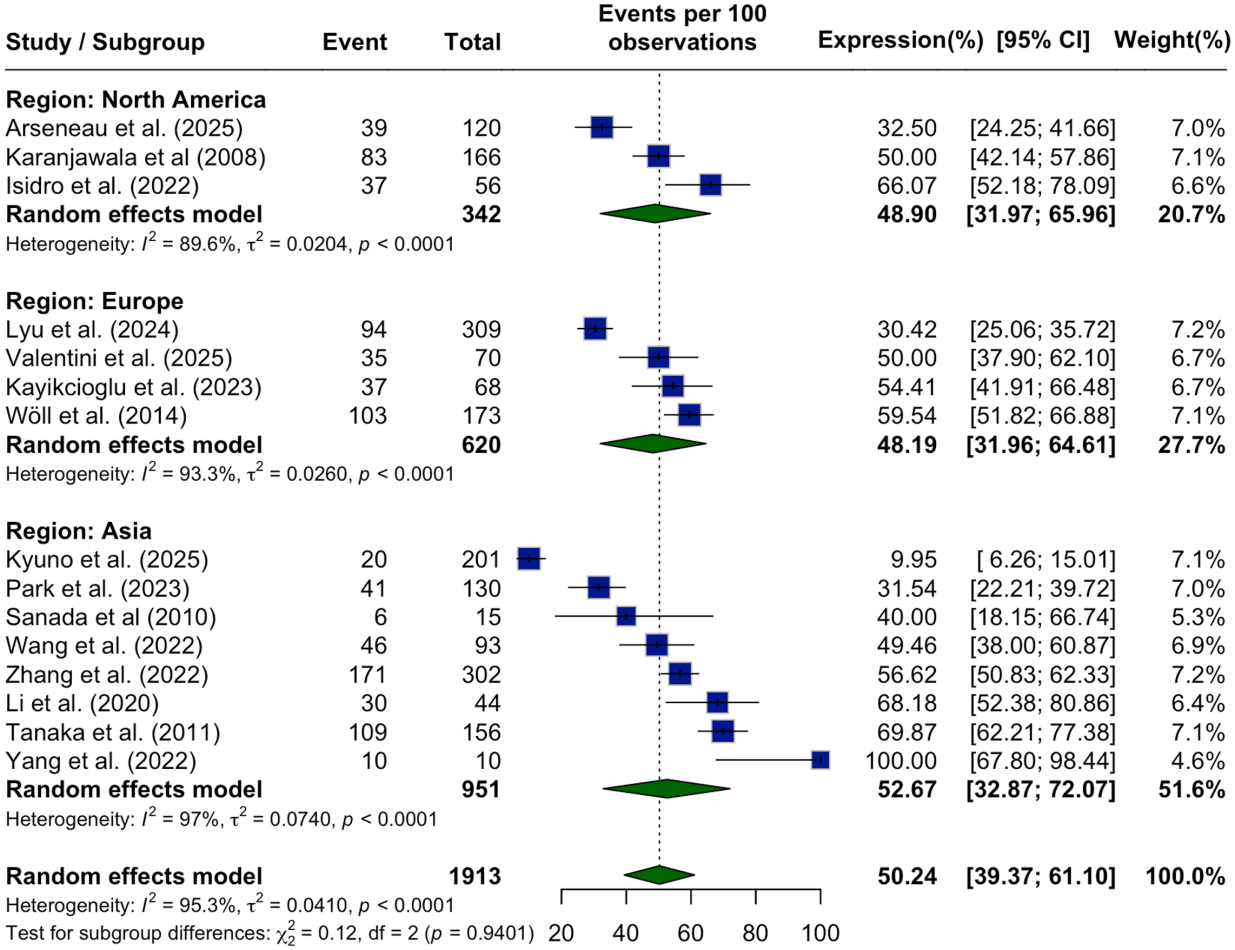
Subgroup analysis of CLDN18.2 expression by geographic region shown as a forest plot stratified by study location. Pooled prevalence estimates were highest in Asia (52.67%), followed by North America (48.90%), and Europe (48.19%), with no statistically significant differences between subgroups (*p* = 0.9401).

The pooled prevalence of CLDN18.2 expression in PDAC was 51.60% (95% CI: 40.93–62.19%) under a random-effects model, with substantial heterogeneity observed across studies (I^2^ = 95.4%, *p* < 0.0001; Figure 2). Subgroup analyses were conducted to explore sources of heterogeneity in CLDN18.2 expression among patients with PDAC. When stratified by geographic region, the pooled expression rates were relatively consistent across continents but demonstrated notable heterogeneity within each subgroup. Studies conducted in Asia reported a pooled expression rate of 52.67% (95% CI: 32.87–72.07%), which was slightly higher than those.

from Europe (48.19, 95% CI: 31.96–64.61%) and North America (48.90, 95% CI: 31.97–65.96%). Despite these variations, no statistically significant difference in CLDN18.2 expression was observed across regions (*p* = 0.9401), and high levels of heterogeneity persisted within each subgroup (Asia: I^2^ = 97.0%, Europe: I^2^ = 93.3%, North America: I^2^ = 89.6%; Figure 3).

**Figure 4:**
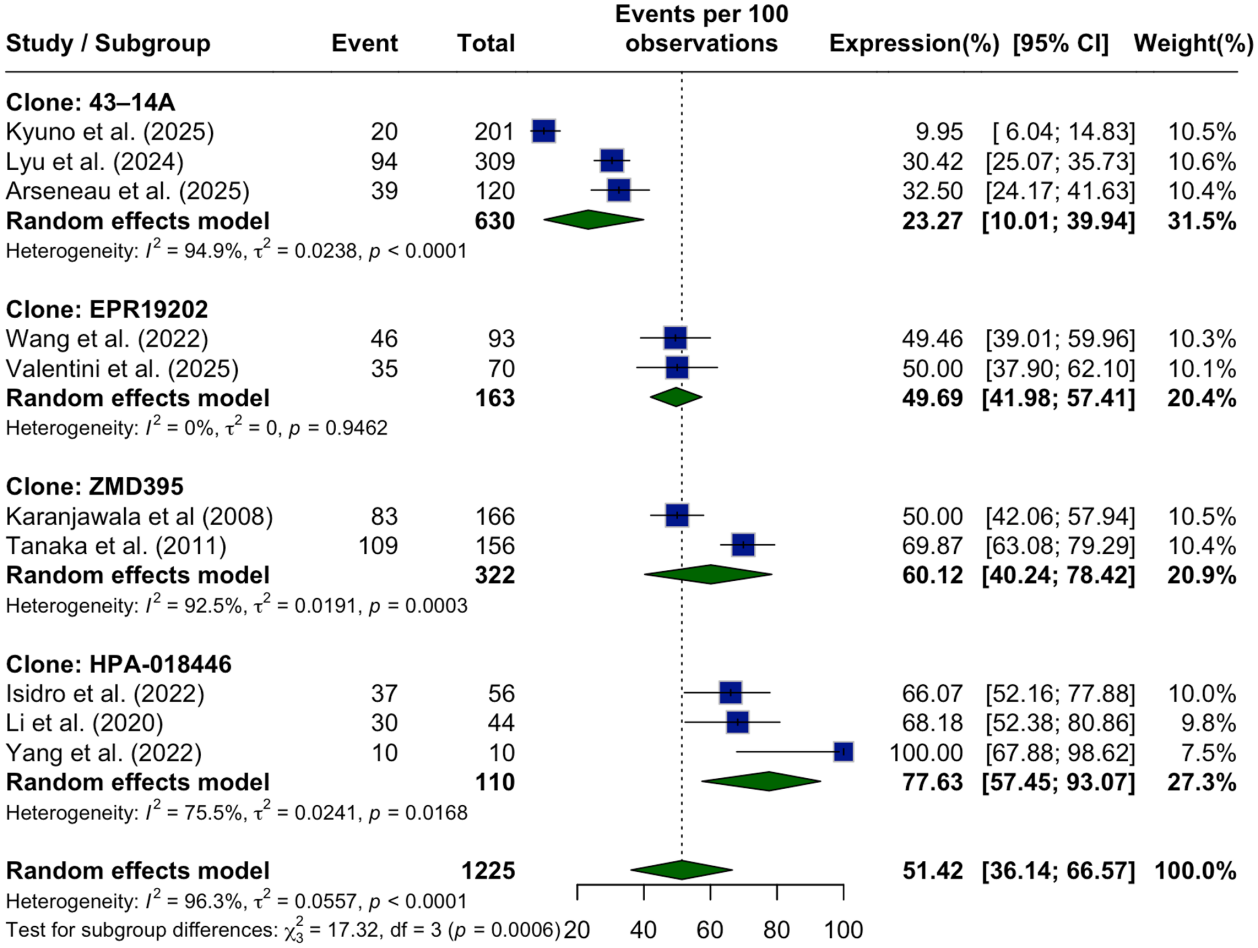
Subgroup analysis of CLDN18.2 expression by antibody clone shown as a forest plot comparing pooled prevalence estimates across assays. Expression was lowest with clone 43–14A (23.27%) and highest with HPA-018446 (77.63%), with statistically significant differences observed between clones (*p* = 0.0006), indicating assay-related variability.

A separate subgroup analysis was performed based on the antibody clone used for IHC detection of CLDN18.2. Considerable differences in expression rates were observed across antibody clones. Studies using the 43–14A clone reported the lowest pooled expression rate at 23.27% (95% CI: 10.01–39.94%), while those employing the HPA-018446 clone reported the highest rate at 77.63% (95% CI: 57.45–93.07%). Intermediate expression rates were observed with the EPR19202 clone (49.69, 95% CI: 41.98–57.41%) and the ZMD395 clone (60.12, 95% CI: 40.24–78.42%). The test for subgroup differences revealed a statistically significant difference in CLDN18.2 expression depending on the antibody clone used (*p* = 0.0006), as shown in Figure 4. These findings emphasize the importance of methodological consistency and antibody selection in the assessment of CLDN18.2 expression across studies.

**Figure 5:**
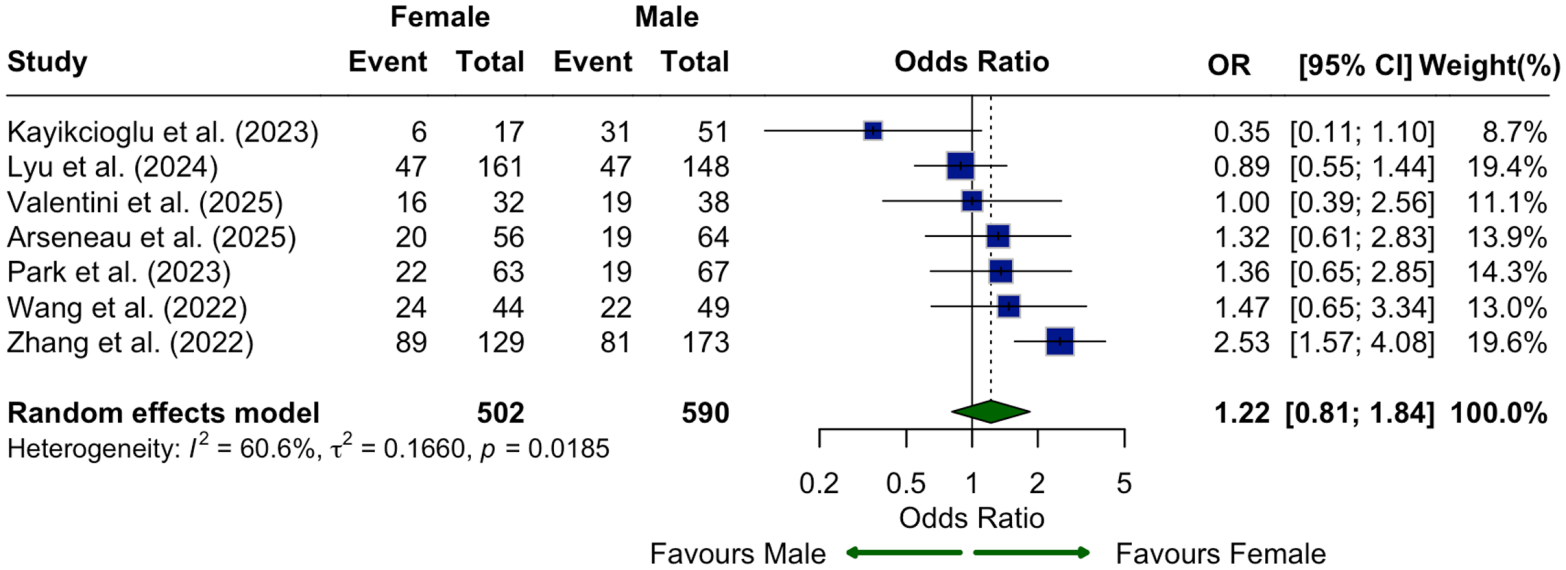
Association between CLDN18.2 expression and patient sex shown as a forest plot of odds ratios comparing male and female patients. No statistically significant association was observed (OR, 1.22; 95% CI, 0.81–1.84), with moderate heterogeneity (*I*^2^ = 60.6%).

Seven studies compared CLDN18.2 expression between male and female patients. The pooled odds ratio (OR) slightly favored higher expression in females (OR = 1.22, 95% CI: 0.81–1.84), though this was not statistically significant (Figure 5).

**Figure 6:**
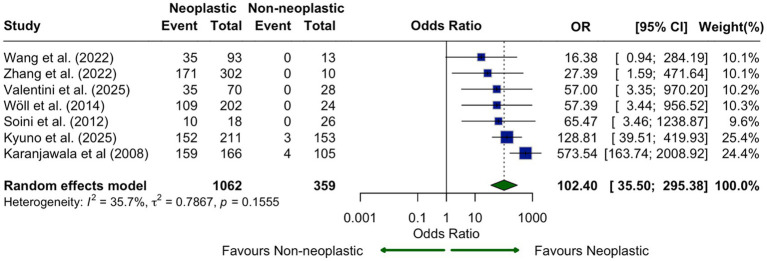
CLDN18.2 expression in neoplastic versus non-neoplastic pancreatic tissue shown as a forest plot comparing odds ratios across seven studies. CLDN18.2 was significantly overexpressed in neoplastic tissue compared with adjacent non-tumor tissue (OR, 102.40; 95% CI, 35.50–295.38), supporting tumor-specific expression.

In six studies that compared CLDN18.2 expression between neoplastic and non-neoplastic pancreatic tissues, expression was significantly higher in neoplastic tissues (OR = 102.40, 95% CI: 35.50–295.38, I^2^ = 35.7%, *p* = 0.1555), indicating tumor-specific expression (Figure 6). Studies such as Karanjawala et al. and Kyuno et al. demonstrated near-complete absence of CLDN18.2 in normal tissue.

**Figure 7:**
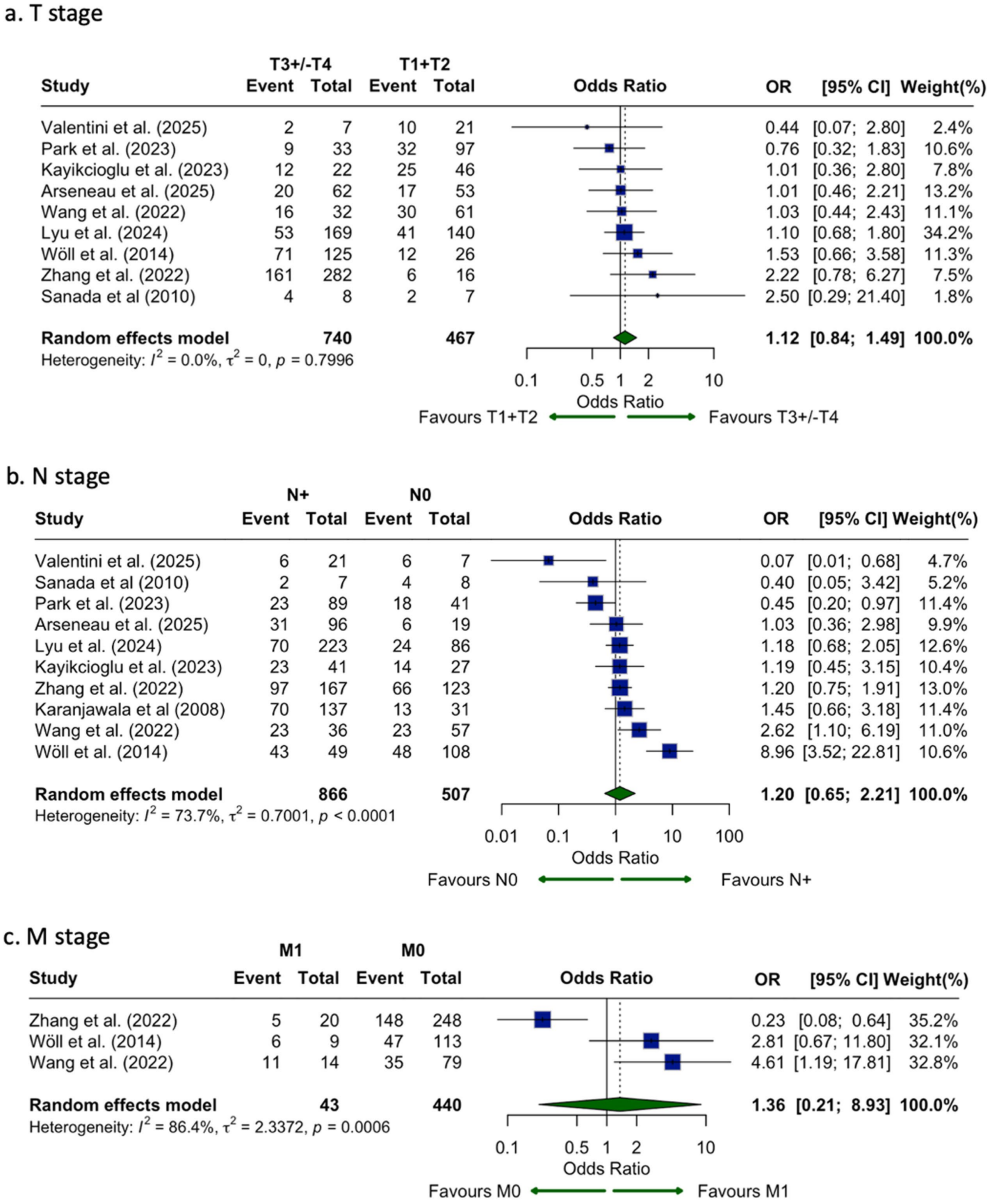
Association of CLDN18.2 expression with tumor stage **(a)**, nodal status **(b)**, and distant metastasis **(c)** shown as forest plots of odds ratios. No statistically significant associations were observed for T stage (T3/T4 vs. T1/T2; OR, 1.12; 95% CI, 0.84–1.49), nodal involvement (N + vs. N0; OR, 1.20; 95% CI, 0.65–2.21), or distant metastasis (M1 vs. M0; OR, 1.36; 95% CI, 0.21–8.93).

CLDN18.2 expression did not significantly differ between early (T1–T2) and advanced (T3–T4) stages (OR = 1.12, 95% CI: 0.84–1.49; I^2^ = 0%; Figure 7a). Similarly, no statistically significant difference was observed between CLDN18.2 expression and nodal status (OR = 1.20, 95% CI: 0.65–2.21; I^2^ = 73.7%; Figure 7b). Three studies evaluated the correlation between CLDN18.2 expression and M stage. The pooled analysis suggested higher expression in metastatic disease (OR = 1.36, 95% CI: 0.21–8.93; I^2^ = 86.4%; Figure 7c), but results were not statistically significant.

**Figure 8:**
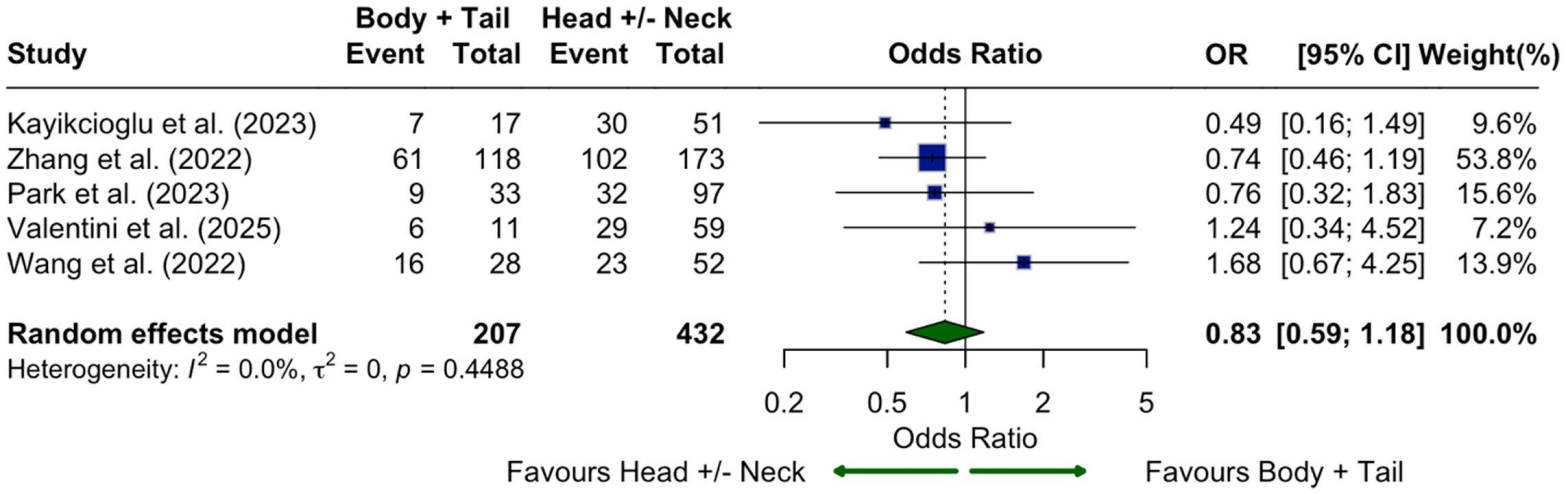
Association of CLDN18.2 expression with tumor location within the pancreas shown as a forest plot comparing head and neck versus body and tail tumors. No statistically significant difference was observed (OR, 0.83; 95% CI, 0.59–1.18), with no observed heterogeneity (I^2^ = 0.0%).

**Figure 9:**
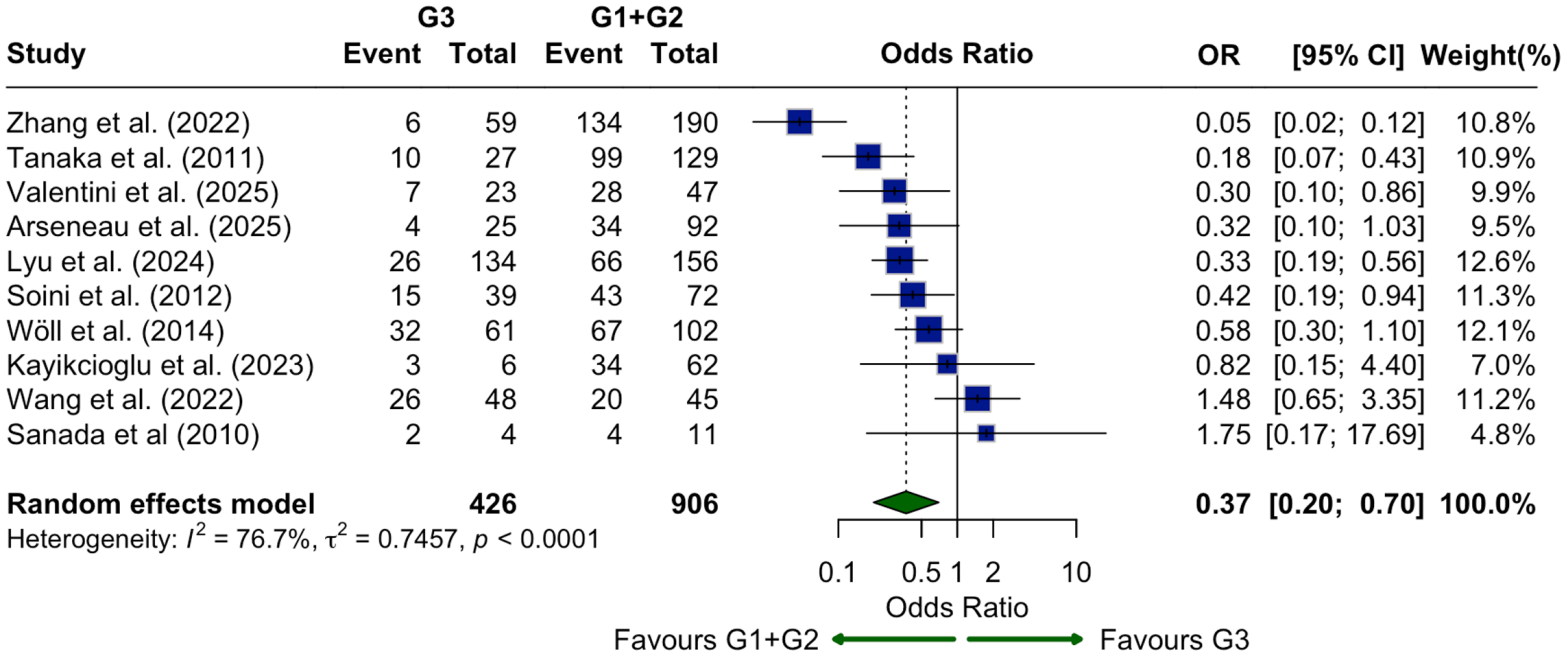
Association of CLDN18.2 expression with tumor grade shown as a forest plot comparing poorly differentiated (G3) tumors with well or moderately differentiated (G1/G2) tumors. CLDN18.2 expression was significantly lower in G3 tumors (OR, 0.37; 95% CI, 0.20–0.70; *p* < 0.0001).

No significant association was found between CLDN18.2 expression and tumor location (body/tail vs. head/neck; OR = 0.83, 95% CI: 0.59–1.18; I^2^ = 0%; Figure 8). Ten studies assessed CLDN18.2 in relation to tumor differentiation. Expression was significantly higher in well or moderately differentiated tumors compared to poorly differentiated ones (OR = 0.37, 95% CI: 0.20–0.70; I^2^ = 76.7%; Figure 9), supporting its association with favorable histology.

**Figure 10:**
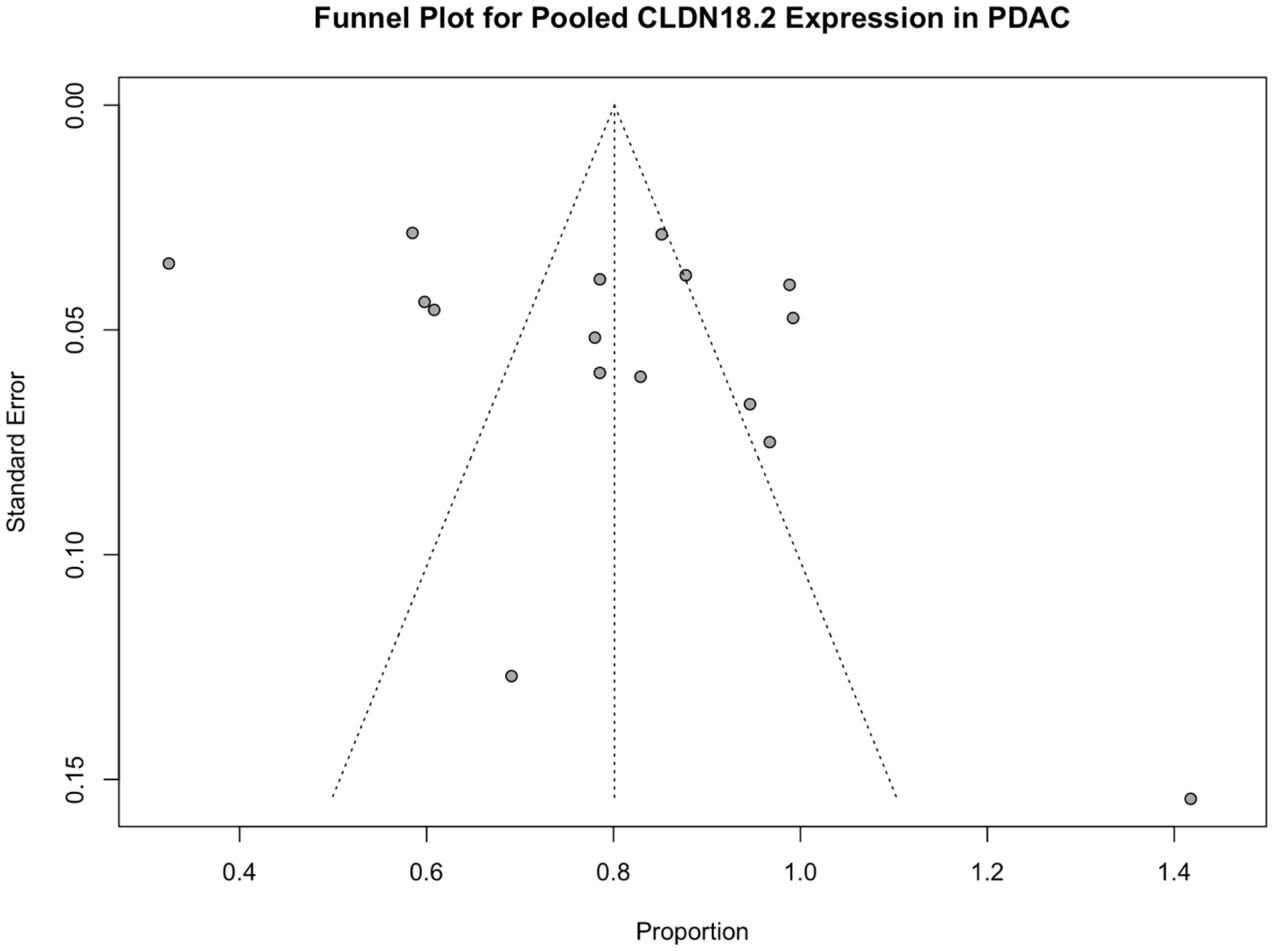
Funnel plot assessing potential publication bias in studies reporting pooled CLDN18.2 expression in pancreatic ductal adenocarcinoma (PDAC). The distribution shows mild asymmetry, suggesting possible small-study effects.

Visual inspection of the funnel plot demonstrated mild asymmetry, which may reflect small-study effects rather than clear publication bias (Figure 10).

## 4 Discussion

### 4.1 Clinical context and rationale

Pancreatic ductal adenocarcinoma remains one of the most lethal malignancies globally, largely due to its late-stage diagnosis and limited responsiveness to current therapies (13, 14). Despite advances in surgical techniques and systemic treatments, survival outcomes remain poor, with minimal progress over recent decades. The emergence of CLDN18.2 as a therapeutic target in gastric and gastroesophageal adenocarcinomas which was highlighted by the success of zolbetuximab in the SPOTLIGHT and GLOW trials [12], has prompted investigation into its expression and relevance in PDAC. This meta-analysis aimed to synthesize current evidence on CLDN18.2 expression in PDAC, evaluate its clinicopathological associations, and explore its potential as a biomarker and therapeutic target.

### 4.2 Methodological variability and assay standardization

This meta-analysis demonstrates that CLDN18.2 is expressed in approximately half of PDAC cases, with a pooled prevalence of 51.6%, and is largely absent from non-neoplastic pancreatic tissue (15–17), reinforcing its tumor-specific nature. However, substantial variability in reported prevalence across studies was observed, primarily driven by differences in antibody clones and staining criteria. Studies applying strict, trial-aligned thresholds (e.g., ≥75–80% moderate/strong membranous staining using clones like 43–14A or LS B16145) consistently reported lower, clinically relevant prevalence estimates, aligning with eligibility criteria used in CLDN18.2-targeted trials. In contrast, studies using permissive cut-offs—such as any detectable staining or low H-scores—tended to overestimate prevalence. These findings underscore the importance of standardized IHC protocols, including validated antibody clones and scoring systems, particularly for guiding patient selection in clinical trials.

### 4.3 Clinicopathological and prognostic significance of CLDN18.2 expression in PDAC

Analysis of clinicopathological variables revealed that CLDN18.2 expression was not significantly associated with tumor stage, nodal status, metastasis, tumor location, or patient sex. These findings suggest that CLDN18.2 expression may be relatively stable across disease extent and anatomical site. However, a notable exception was tumor grade: expression was significantly lower in poorly differentiated tumors (G3) compared to well or moderately differentiated ones (G1/G2), with a pooled OR of 0.37. This reduction may reflect the loss of epithelial characteristics in high-grade tumors, consistent with the known association between CLDN18.2 and epithelial integrity (13, 15, 18). Given this relationship with tumor differentiation, it is important to determine whether CLDN18.2 expression has prognostic relevance. However, current evidence remains inconclusive: Park et al. (13), reported no association with survival after excluding metastatic cases, whereas Lyu et al. (18), observed improved overall survival in CLDN18.2-positive patients (30 vs. 18 months).

### 4.4 Tumor specificity of CLDN18.2 expression

CLDN18.2 expression is highly specific to neoplastic pancreatic tissue (pooled OR ≈ 102 vs. non-neoplastic tissue), making it an attractive biomarker for targeted therapy with the potential for low off-tumor toxicity and improved therapeutic precision. In addition to its therapeutic relevance, this tumor-restricted expression profile also supports the use of CLDN18.2 as a molecular imaging target, offering the prospect of strong tumor uptake with minimal background signal in normal tissues. Notably, several CLDN18.2-targeted PET tracers are currently under investigations (19).

### 4.5 Cross-cancer context and expression benchmarking

Comparing CLDN18.2 expression in PDAC with other tumor types offers useful context for therapeutic development. In the SPOTLIGHT and GLOW trials, CLDN18.2 expression was reported in 41% of gastric cancers and 37.3% of gastroesophageal junction (GEJ) adenocarcinomas using strict IHC positivity thresholds (17). In our meta-analysis, the overall pooled prevalence in PDAC was slightly higher, although heterogeneity in assay methodology limits direct comparison. When restricting the analysis to studies using the same IHC protocol as SPOTLIGHT/GLOW, the pooled PDAC expression was lower (23.3%). Although the subset analysis using SPOTLIGHT/GLOW criteria suggests lower CLDN18.2 expression in PDAC than in gastric and GEJ cancers, comparable variability driven by staining thresholds and tumor characteristics has also been reported in gastric cancer cohorts (20). Moreover, an expression rate of ~23% is still clinically meaningful given the overall scarcity of effective treatment options and the persistently poor prognosis associated with PDAC. These findings support the continued investigation of CLDN18.2 as a therapeutic target in PDAC and highlight the importance of harmonized assessment protocols to enable reliable cross-cancer comparisons.

### 4.6 Therapeutic implications of CLDN18.2 and evidence from preclinical models

The therapeutic relevance of CLDN18.2 in PDAC is further supported by preclinical evidence. Zolbetuximab has shown potent cytotoxic activity against both endogenous and transduced CLDN18.2-expressing pancreatic cancer cells, particularly when combined with chemotherapy agents such as gemcitabine (21). The effect of neoadjuvant chemotherapy on CLDN18.2 expression, however, appears variable: while some experimental studies report increased expression following gemcitabine-based treatment that may enhance target availability (21), Lyu et al. observed reduced expression after neoadjuvant chemotherapy, although the specific regimen was not reported (18). These divergent findings may reflect differences in chemotherapy agents, tumor biology, or IHC methodology, and collectively highlight the need for timepoint-specific assessment of CLDN18.2 expression in multimodal treatment settings. Together, these data provide a strong rationale for ongoing clinical development of CLDN18.2-targeted therapies in PDAC, including zolbetuximab (NCT03816163; NCT06396091) and emerging modalities such as CLDN18.2-directed CAR-T cells. Standardized and biologically informed patient selection will be essential to maximizing clinical benefit as these therapies progress.

### 4.7 Limitations and future directions

While this meta-analysis offers the most comprehensive synthesis to date on CLDN18.2 expression in PDAC, several limitations must be acknowledged. First, substantial heterogeneity across studies—particularly in antibody clones, staining protocols, and positivity thresholds—limits cross-study comparability and may confound prevalence estimates. Second, survival outcomes and treatment responses were inconsistently reported, precluding a robust evaluation of prognostic and predictive value. Third, some subgroup analyses (e.g., by metastatic status or specific clones) were based on a small number of studies, reducing statistical power. Future research should focus on developing and validating standardized IHC assays aligned with clinical trial criteria (e.g., SPOTLIGHT/GLOW), and on conducting prospective, biomarker-driven trials to evaluate the role of CLDN18.2 in patient selection, therapy monitoring, and long-term outcomes. Exploration of its expression in precursor lesions and across treatment timepoints could further inform its utility in disease stratification and early intervention.

## 5 Conclusion

This meta-analysis highlights CLDN18.2 as a promising tumor-specific biomarker in pancreatic ductal adenocarcinoma, expressed in over half of cases and absent in non-neoplastic pancreatic tissue. Notably, even under strict positivity thresholds aligned with current clinical trial criteria, almost one-fifth of PDAC patients would qualify for CLDN18.2-targeted therapy. Although not significantly associated with most clinicopathological variables, its reduced expression in poorly differentiated tumors suggests potential prognostic value. The marked variability in reported prevalence—driven by differences in antibody clones and scoring criteria—underscores the urgent need for assay standardization. Preclinical data and parallels with gastric cancer support the feasibility of CLDN18.2-targeted therapies in PDAC, with several ongoing trials exploring this potential. Future research should prioritize unified diagnostic protocols, prospective clinical validation, and exploration of CLDN18.2 as both a predictive and dynamic biomarker to guide precision therapy in pancreatic cancer.

## Data Availability

The original contributions presented in the study are included in the article/supplementary material, further inquiries can be directed to the corresponding author.
